# Electronegativity Assisted Synthesis of Magnetically Recyclable Ni/NiO/g-C_3_N_4_ for Significant Boosting H_2_ Evolution

**DOI:** 10.3390/ma14112894

**Published:** 2021-05-28

**Authors:** Tingfeng Zhang, Ping Liu, Lili Wang, Shuai Wang, Jinsheng Shi, Xuefang Lan

**Affiliations:** 1 Department of Chemistry and Pharmacy, Qingdao Agricultural University, Qingdao 266000, China; ztf824066980@163.com (T.Z.); pingliu@qau.edu.cn (P.L.); liliwang@qau.edu.cn (L.W.); jsshiqn@aliyun.com (J.S.); 2 Chengyang branch of Qingdao Ecological Environment Bureau, Qingdao 266000, China; cdws@163.com

**Keywords:** Ni/NiO/g-C_3_N_4_, magnetically recoverable, ethanol-solvothermal, environmental-friendly synthesis

## Abstract

A magnetically recyclable Ni/NiO/g-C_3_N_4_ photocatalyst with significantly enhanced H2 evolution efficiency was successfully synthesized by a simple ethanol-solvothermal treatment. The presence of electronegative g-C_3_N_4_ is found to be the key factor for Ni^0^ formation in ternary Ni/NiO/g-C_3_N_4_, which provides anchoring sites for Ni^2+^ absorption and assembling sites for Ni^0^ nanoparticle formation. The metallic Ni^0^, on one side, could act as an electron acceptor enhancing carrier separation and transfer efficiency, and on the other side, it could act as active sites for H_2_ evolution. The NiO forms a p–n heterojunction with g-C_3_N_4_, which also promotes carrier separation and transfer efficiency. The strong magnetic property of Ni/NiO/g-C_3_N_4_ allows a good recyclability of catalyst from aqueous solution. The optimal Ni/NiO/g-C_3_N_4_ showed a full-spectrum efficiency of 2310 μmol·h^−1^·g^−1^ for hydrogen evolution, which is 210 times higher than that of pure g-C_3_N_4_. This ethanol solvothermal strategy provides a facile and low-cost synthesis of metal/metal oxide/g-C_3_N_4_ for large-scale application.

## 1. Introduction

Photocatalysis has attracted great attention in environmental protection and the domain of new energy application as it is an effective technique to degrade water pollutants and convert sustainable solar energy into applicable chemical energy, such as hydrogen. Carbon nitride, as a metal-free polymer material, has been widely used in photocatalytic water splitting since 2009, due to its advantages of non-toxicity, low cost, high stability, excellent optical properties, and electronic structure [1,2,3]. Nevertheless, the low separation and high recombination efficiency of photogenerated charge carriers limits its application in H_2_ evolution. Loading noble metals such as Au, Ag, and Pt onto carbon nitride as co-catalysts is an effective way to solve these problems. However, the high price of noble metals limits their application in practical conditions. As a transition metal, nickel and nickel-containing compounds are considered to be an effective co-catalyst for photocatalysis [4]. 

Many methods have been reported to load nickel species onto g-C_3_N_4_. For example, Ni/NiO core-shell particles can be loaded on a g-C_3_N_4_ nanosheet by using a high temperature hydrogen reduction method (H_2_, 200–400 °C), which greatly improved the hydrogen evolution efficiency under visible light irradiation [5]. Amorphous NiO prepared by high temperature calcination (air, 300 °C) can form heterojunction with g-C_3_N_4_, hence improving the efficiency of photocatalytic hydrogen production [6]. The Ni nanoparticles that loaded onto sulfur-doped g-C_3_N_4_ nanosheets by photodeposition can be used as the active center to participate in hydrogen production [7]. These works reveal the advantages of nickel-based materials for photocatalytic hydrogen production, such as good stability, high activity, and earth abundance. Nevertheless, most of these photocatalysts still suffer disadvantages, e.g., the harsh and dangerous synthetic conditions or the single phase obtained in one method. Additional papers related to Ni/g-C_3_N_4_ or NiO/g-C_3_N_4_ composite synthesized by different methods are summarized in Table S1. Compared with these articles, our preparation method not only avoided harsh preparation conditions such as high reduction temperature under H_2_ atmosphere, but also constructed magnetic recoverable Ni/NiO/g-C_3_N_4_ ternary composite in one step, which has not previously been reported.

The magnetic recoverability of photocatalysts is of key importance in practical applications. Therefore, many studies have focused on designing magnetic composites for practical photocatalysts. Core-shell catalysts like Fe_3_O_4_@ZnS and NiCo_2_O_4_@ZnS [8] have been synthesized using surfactant to build the magnetic properties of photocatalysts. However, the high cost of surfactant limits its further application in practice. g-C_3_N_4_/graphene/NiFe_2_O_4_ [9] and ZnFe_2_O_4_-Graphene [10] were synthesized using a hydrothermal strategy to establish their magnetic properties. However, only metal oxides (NiCo_2_O_4_, Fe_3_O_4_) instead of metallic metal (e.g., Ni, Co, Fe) that can be introduced by hydrothermal strategy for photocatalysts were used.

In this work, a Ni/NiO/g-C_3_N_4_ composite was first constructed by a one-step solvothermal strategy. The Ni and NiO can be composited with g-C_3_N_4_ simultaneously by a mild solvothermal treatment (160 °C) using ethanol as solvent. The NiO forms a p–n heterojunction with g-C_3_N_4_ [11]; the metallic Ni^0^, on one side, act as an electron acceptor, lowering the recombination photogenerated charge carriers, and on the other side, act as active sites for H_2_ evolution. More importantly, the Ni/NiO/g-C_3_N_4_ composite shows strong magnetic properties, which contributes to an easy recoverability of the catalysts. The optimum catalyst showed a full-spectrum efficiency of 2310 μmol·h^−1^·g^−1^ in photocatalytic H_2_ production, which is 210 times higher than that of bulk carbon nitride.

Compared with hydrogen reduction under high temperature [5], our ethanol-solvothermal-strategy (160 °C) avoids the use of dangerous H_2_ and high temperature during preparation, and can introduce Ni/NiO onto g-C_3_N_4_ in one step. This solvothermal preparation method can be used as a universally applicable strategy for Metal/Metal-oxide/g-C_3_N_4_ composites. Through a series of controlled trials, we have confirmed that the electronegativity of g-C_3_N_4_ is a key factor for the formation of Ni^0^.

## 2. Experimental Section

### 2.1. Materials

Nickel acetate tetrahydrate (Ni(CH_3_COO)_2_·4H_2_O, ≥98.0%, Sigma Aldrich), urea ((NH_2_)2CO, ≥99.5%, Sigma Aldrich), triethanolamine ((C_2_H_5_O)_3_N, ≥99.0%, Sigma Aldrich), and ethanol (C_2_H_5_OH, ≥99.7%, China National Medicines Corporation Ltd., Beijing, China) were used in experiments without further purification.

### 2.2. Preparation of Bulk g-C_3_N_4_

Bulk g-C_3_N_4_ was synthesized by thermal polymerization method. In a typical synthesis process, 10 g of urea was put in a crucible with cover holds and heated in a muffle furnace at 550 °C for 4 h with a heating rate of 10 °C min^−1^. The obtained products, after calcination and grounding, were collected for further use.

### 2.3. Preparation of Ni/NiO/g-C_3_N_4_ Samples

**Figure 1:**
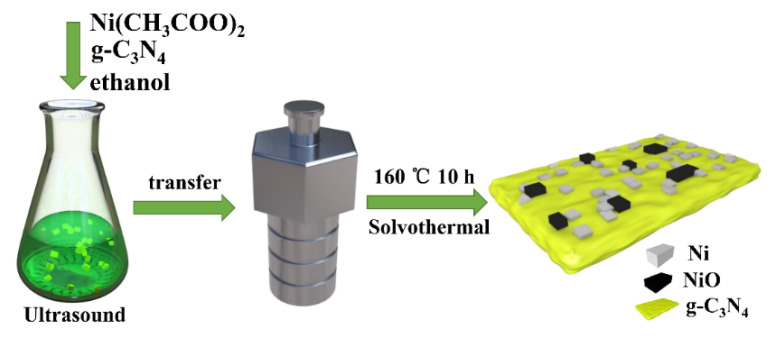
The synthetic route of Ni/NiO/g-C_3_N_4_ composite.

The Ni/NiO/g-C_3_N_4_ samples were synthesized by a simple solvothermal method as shown in Figure 1. In detail, the bulk carbon nitride (30 mg) was dispersed in 15 mL anhydrous ethanol and subjected to ultrasound for 60 min. Afterwards, 30 mg of Ni(CH3COO)2 was added to the solution and subjected to ultrasonic treatment for another 30 min. The solution was then transferred into a 20 mL Teflon-lined autoclave and heated at 160 °C for 10 h to form the final samples. After being naturally cooled to room temperature, the prepared sample was washed with ethanol three times and collected by centrifugation. The resulting product was vacuum dried overnight at 60 °C and named 1.0 Ni/CN. The weight ratio of nickel acetate to carbon nitride in precursor was 0.4, 0.6, 0.8, 1 and 1.2, respectively; therefore, the synthesized samples were named x-Ni/CN (x = 0.4, 0.6, 0.8, 1 and 1.2). Ni(CH_3_COO)_2_ without the addition of g-C_3_N_4_ was also prepared with solvothermal treatment to prepare the controlled sample trials, and the resulting product was named Ni/0CN.

The synthesis process of Ni/GO is similar to that of x-Ni/CN, except that the solvothermal reaction is carried out by replacing g-C_3_N_4_ with the same amount of GO (i.e., graphene oxide). The Mg/Ni/CN were prepared according to the same preparation procedure of x-Ni/CN, with different amounts of Mg(CH_3_COO)_2_ added to the precursor solution together with the addition of Ni(ac)_2_. The molar ratio of Mg/Ni is 2 and 1.

The protonated H+/CN was prepared by soaking and heating g-C_3_N_4_ in 1 mol/L HCl at 80 °C for 10 h.

### 2.4. Characterization

The X-ray diffraction (XRD) patterns were tested with D8 diffractometer (Cu-Kα radiation, λ = 0.15406 nm) agent produced by Germany Bruker AXS Co. Ltd., (karlsruh, Germany). The X-ray photoelectron spectroscopy (XPS) was determined on an ESCA-3 Mark II spectrometer (VG Scientific Ltd., Devon, England) using Al Ka (1486.6 eV) radiation. The Hitachi HT-7700 instrument was used for the measurement of transmission electron microscopy (TEM) with an acceleration voltage of 200 kV. The general morphology of the photocatalyst was obtained by scanning electron microscopy (SEM, 7500F, JEOL) equipped for energy-dispersive X-ray (EDX) spectroscopy. Ultraviolet–visible (UV–vis) diffuse reflectance spectrum (DRS) spectra were recorded on a UV–vis spectrophotometer (Beijing Purkay General Instrument Co. Ltd., TU-1901, Beijing, China). The Brunauer–Emmett–Teller (BET) surface areas of the synthesized samples were measured with a Quantachrome N22–27E analyzer at 77 K. Photoluminescence (PL) spectra of photocatalysts were recorded on a Hitachi F-4600 fluorescence spectrophotometer. The synthesized samples were measured by a Fourier transform infrared (FTIR) spectrometer (Thermo Fisher Scientific, Nicolet IS5, Waltham, MA, USA) at room temperature. The N2 adsorption–desorption isotherm were analyzed on a Micromeritics ASAP 2020 instrument (Micromeritics Instrument Co., Norcross, GR, USA). The BET surface areas were calculated using the Barrett–Joyner–Halenda (BJH) method. ATR-FTIR spectra were recorded on an infrared spectrometer (Thermo Fisher Scientific, Nicolet IS5, Waltham, MA, USA).

### 2.5. Photoelectrochemical Measurements

The photoelectrochemical measurements were performed using a CHI 760E electrochemical workstation. The standard three-electrode appliance was used to determine the electrochemical impedance spectra (EIS) measurement, in which the calomel electrode was used as the reference electrode and the counter electrode was the Pt electrode. 0.5 M Na_2_SO_4_ aqueous solution was utilized as the electrolyte. A 300 W Xenon lamp (PLS-SXE 300, Beijing Bofeilai Co., Beijing, China) was used as the light source. When the baseline of the electrochemical workstation was stabilized, EIS measurements were conducted in the frequency range of 10^5^–10^−1^ Hz at open circuit potential with an alternating current voltage amplitude of 20 mV. Transient photocurrent was accomplished in a visible light irradiation system that continuously cycled on/off.

The working electrodes were prepared as follows: In 0.5 mL of isopropyl alcohol, 0.05 g of each of the photocatalysts was ultrasonically dispersed to form a homogeneous mixture. Then, the mixture was poured onto a 5 cm × 1 cm fluorine-doped tin oxide (FTO) glass electrode.

### 2.6. Photocatalytic Activity Evaluation

#### 2.6.1. Photocatalytic Hydrogen Production Test

The photocatalytic hydrogen production experiment was performed in a vacuum quartz reactor with a cooling water system (LX-300, Beijing Zhongjiaojinyuan Co., Beijing, China). Typically, 15 mg of the catalyst powders was dispersed in a 50 mL aqueous solution containing 10 mL of triethanolamine (TEOA, 10 vol. %, as a sacrificial reagent). Before light irradiation, air in a quartz reactor and dissolved oxygen in liquid solution were extracted by vacuum pump. A 300 W Xenon lamp without any filters (PLS-SXE 300, Beijing Bofeilai Co., Beijing, China) was used as the light source. The hydrogen production was evaluated every one hour using an online gas chromatography (GC7900, Techcomp, Shanghai, 5A molecular sieve column) with a TCD detector. The test was carried out at a column temperature of 40 °C, using nitrogen as the carrier gas.

#### 2.6.2. Photocatalytic Degradation Experiment

As a widely used organic dye, rhodamine B (RhB) was chosen as a pollutant for the photocatalytic degradation experiment. A 300 W Xenon lamp without any filters (PLS-SXE 300, Beijing Bofeilai Co., Beijing, China) was used as the light source. Prior to light irradiation, a 30 mg sample was dispersed into the RhB solution (100 mL, 5 mg/L) and stirred under dark conditions for 30 min to allow an adsorption–desorption equilibrium to be achieved between the photocatalysts and RhB. Upon starting light irradiation, 5 mL of solution was sampled and centrifuged every 15 min to collect the upper liquid and test the dye concentration. Finally, the concentration of RhB was measured using a TU-1901 spectrophotometer

## 3. Results and Discussion

### 3.1. Ni/NiO/g-C_3_N_4_ Formation Mechanism

**Figure 2:**
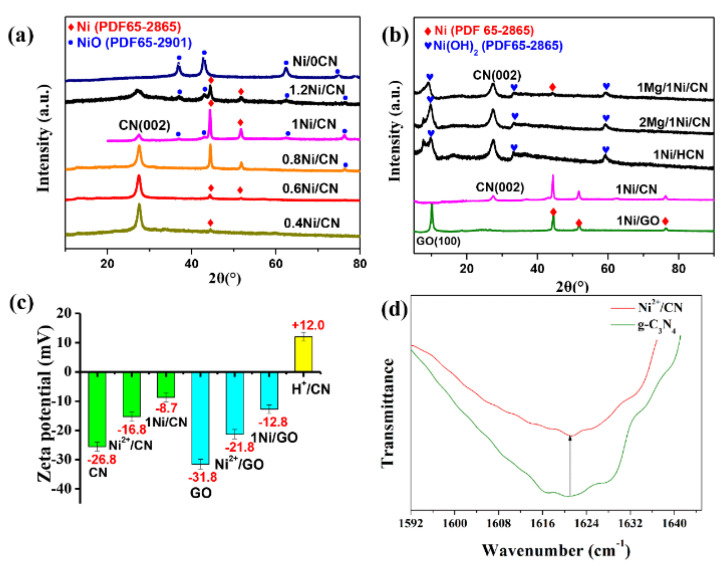
(**a**) XRD patterns of x-Ni/CN samples, (**b**) XRD patterns of Mg/Ni/CN and Ni/GO, (**c**) analysis of zeta potential on the surface of g-C_3_N_4_ and GO, and (**d**) OH-specific mode at 1621 cm^−1^ for g-C_3_N_4_ and Ni^2+^/CN.

The synthesized x-Ni/CN samples were characterized by XRD to distinguish the phases that formed on each catalyst (Figure 2a). Peaks at 27.5° were assigned to the (002) peak of g-C_3_N_4_ caused by the inter-layer accumulation of the g-C_3_N_4_ aromatic layer [12]. Peaks at 44.5° and 51.8° are due to the Ni crystal phase (JCPDS No. 65-2865); and peaks at 37.1°, 43.1°, 62.6°, and 75.0 are related to the formation of NiO (JCPDS No.65-1049). As the Ni/CN ratio increased from 0.4 to 1.0, the Ni peaks became more intense indicating the increasing reduction of Ni(ac)_2_ to Ni by ethanol solvent; the g-C_3_N_4_ peak became weaker likely because the new-formed Ni/NiO species covers g-C_3_N_4_ in samples. Once the Ni/CN ratio further increased from 1.0 to 1.2, the Ni peaks became weaker, whereas, on the other hand, the NiO peaks became more intense. This suggests that overloading of Ni in the precursor solution results in the formation of NiO due to the limited reduction capability of ethanol solvent. Note that, if g-C_3_N_4_ was not added to the precursor solution, only NiO could be obtained on the final catalysts (Figure 2a). This suggests that the presence of g-C_3_N_4_ is the crucial factor for Ni^0^ formation in this preparation.

Since Ni^0^ are critical species for photocatalytic H_2_ evolution due to its electron transfer accelerator and active sites effects, and Ni^0^ can only be formed under the presence of g-C_3_N_4_ during ethanol solvothermal treatment, it is quite interesting to explore what is the crucial factor for Ni^0^ formation during our preparation.

We considered that the bonding sites of Ni^2+^ provided by electronegative g-C_3_N_4_ are the crucial factor for Ni^0^ formation. To verify our speculation, Mg(CH_3_COO)_2_ (with molar ratio of Mg/Ni = 2:1), which could compete with Ni^2+^ to occupy the electronegative binding sites on g-C_3_N_4_, were added to the precursor solution. As expected, once Mg(CH_3_COO)_2_ with Mg/Ni = 2 was added to the precursor solution, only Ni(OH)_2_ was formed on g-C_3_N_4_ (Figure 2b). The Ni^0^ were not formed as a result of the occupation of electronegative binding sites by Mg^2+^. When lower amounts of Mg(CH_3_COO)_2_ (Mg/Ni = 1) were added, both Ni(OH)_2_ and Ni^0^ could be formed after ethanol solvothermal treatment (Figure 2b). The Ni^0^ formation are due to the available electronegative sites left after Mg^2+^ occupation. These results confirmed that the electronegative sites on g-C_3_N_4_ are crucial for Ni(CH_3_COO)_2_ reduction to Ni^0^.

The crucial effect of the electronegative binding site for Ni^0^ formation was also verified by replacing g-C_3_N_4_ with protonated g-C_3_N_4_ (H^+^/CN) and electronegative GO (Figure 2b). Before protonation treatment, the g-C_3_N_4_ surface was electronegative (zeta potential of −26.8 mV, Figure 2c), and the Ni^0^ was the main phase formed after ethanol treatment, together with a small amount of NiO phase (Figure 2b). Whereas, after protonation treatment, the surface of H^+^/CN showed electropositive properties (zeta potential of +12.0 mV, Figure 2c), the Ni(OH)_2_ instead of Ni^0^ was formed on g-C_3_N_4_ (Figure 2b). On the other hand, if we use electronegative GO as the support in the precursor solution, Ni^0^ were again formed as the main phase after ethanol treatment (Figure 2b).

The zeta potentials of samples after Ni(CH_3_COO)_2_ adsorption and ethanol solvothermal treatment were also measured to confirm the key role of surface electronegativity. The zeta potentials of g-C_3_N_4_, Ni^2+^/g-C_3_N_4_, and 1.0 Ni/CN were measured and the results were determined to be −25.6, −15.3, and −8.7 mV, respectively (Figure 2c), confirming our speculation that electronegative g-C_3_N_4_ provide absorption sites and binding sites for Ni^2+^, and assist Ni^2+^ reduction to Ni^0^ during ethanol solvothermal treatment. Furthermore, the potential of GO, Ni^2+^/GO, and 1.0 Ni/GO (Figure 2b) to follow the same trend of g-C_3_N_4_ confirmed the key function of the electronegative surface for Ni^0^ formation, together with the successful formation of Ni^0^ on the GO support (Figure 2b).

The ATR-IR spectra were also carried out to investigate the functional groups on the surface of g-C_3_N_4_ and Ni^2+^/CN (Figure S1), hence to further explore the Ni^2+^ bonding mechanism on the g-C_3_N_4_ surface. The stretching vibration peak at 3500–3000 cm^−1^ are attributed to N-H stretching mode; the sharp peaks at 1621 cm^−1^ are attributed to the -OH stretching mode of the surface hydroxyls dangling on the g-C_3_N_4_ surface. Ni^2+^ introduction decreases the intensity of N-H and N-H_2_ groups on the surface of g-C_3_N_4_, likely due to the Ni loading on the cavity of the heptazine ring of g-C_3_N_4_ (Figure S2) [13,14], which shades the N-H stretching detected by IR. Furthermore, Ni^2+^ introduction also decrease the intensity of the -OH pattern (Figure 2d), which indicates that the Ni^2+^ species (as Lewis acid) also binds to the surface O^-^ (as Lewis base) deriving from the dissociation of surface hydroxyl groups [15].

Based on the above analysis, we can conclude that the presence of electronegative g-C_3_N_4_ is the key factor for Ni^0^ formation in ternary Ni/NiO/g-C_3_N_4_. The electronegative g-C_3_N_4_ enables Ni^2+^ reduction by providing adsorption sites for Ni^2+^ and binding sites for the Ni^0^ particle. Furthermore, g-C_3_N_4_ may also act as a catalyst facilitating the Ni^2+^ reduction during ethanol-thermal treatment. Acetate may also facilitate Ni^2+^ reduction via its reducibility [16]. Due to the limited reduction capability of ethanol, excessive nickel acetate will decompose into NiO, forming p–n heterojunction with g-C_3_N_4_.

### 3.2. XPS and EDS Analyses

**Figure 3:**
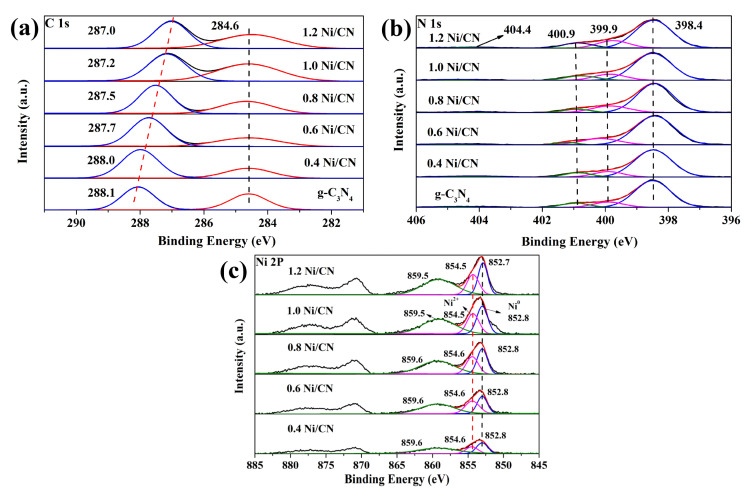
XPS spectra of (**a**) C1s, (**b**) N1s, and (**c**) Ni2p of all the samples.

**Figure 4:**
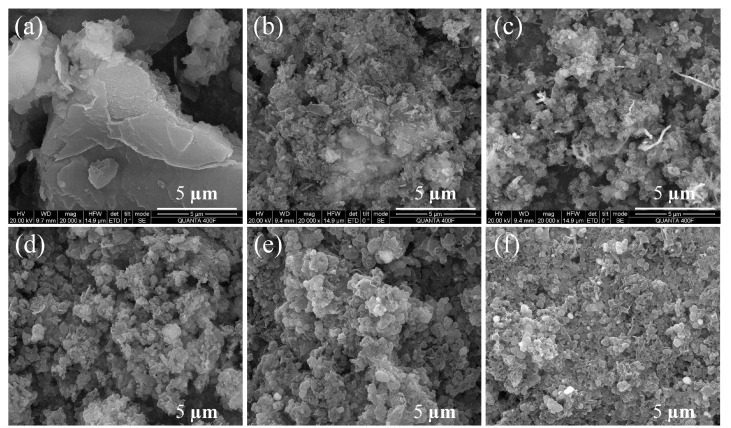
SEM image of (**a**) pure g-C_3_N_4_, (**b**) 0.4 Ni/CN, (**c**) 0.6 Ni/CN, (**d**) 0.8 Ni/CN, (**e**) 1.0 Ni/CN, and (**f**) 1.2 Ni/CN.

To explore the chemical environment of each element in the prepared samples, XPS was carried out (Figure 3a–c). In the C 1 s spectrum (Figure 3a), peak at binding energies of 284.6 eV is due to the sp^2^-hybridized C–C bonds of standard carbon tapes [17]; peak at 288.1 eV is assigned to the sp^2^-bonded carbon atom at the heterocyclic ring (N-C=N) in aromatic carbon nitride, which is the major type of carbon in a triazine-based skeleton [18]. It can be seen from Figure 4a that the peak value at 288.1 eV gradually decreases from 288.1 eV to 287.0 eV as nickel content increases, indicating the increase in electron cloud density of carbon atoms in g-C_3_N_4_, which further proves the strong interaction between Ni and g-C_3_N_4_, i.e., the successful synthesis of the Ni/g-C_3_N_4_ composite. The N 1 s spectrum (Figure 3b) shows two main peaks at 398.4 eV and 399.9 eV, which are assigned to the sp^2^-hybridized nitrogen (C=N-C) and the N-C_3_ groups [19], respectively. Weak peaks at 400.9 eV are also observed due to amino functions (C-N-H) resulting from incomplete condensation of the melon structure [20]. The last faintly visible peak at 404.4 eV is due to the existence of the π-excitation effect [21]. In the N 2p spectrum (Figure 3c), the strongest peak at 852.7 eV is directed to Ni^0^ 2p_3/2_ [7]; the peak at 854.5 eV is assigned to Ni^2+^ 2p_3/2_ of NiO; and the peak at 859.5 eV is ascribed to the satellite signal of Ni^2+^ 2p_3/2_ [4,22]. As the Ni content increases from 0.4 to 1.2, the peak areas of Ni^0^ and Ni^2+^ also increased, indicating the increasing loading of Ni in the composites.

The XPS results confirm a ternary Ni/NiO/g-C_3_N_4_ constitute of composite obtained via our synthesis, which is consistent with XRD results (Figure 2a). Furthermore, energy dispersive (EDS) patterns of samples (Figure S3) show four signal peaks of C, N, O, and Ni, again verifying the coexistence of these elements in the catalyst powders. As the Ni precursor content increases from 0.4, 0.6, and 0.8, to 1.0 and 1.2, the actual loading of Ni increased from 9.57 wt%, 14.55 wt%, and 18.65 wt%, to 23.72 wt% and 29.93 wt%, respectively.

### 3.3. Morphology and Texture Property Analyses

To investigate the structure and morphology of samples, SEM characterizations were carried out (Figure 4 and Figure S4). The pure g-C_3_N_4_ (Figure 4a and Figure S4a) exhibits a regular bulk morphology and a smooth surface with no large pores. As the Ni precursor content was added and increased, the bulk g-C_3_N_4_ disrupted into small particles (Figure 4) with layered and porous structures (zoom-in views in Figure S4). Furthermore, many ball-flower structures were found on the composite surface as the Ni precursor content increased to 1.0 and 1.2 (Figure S4). This is likely because during solvothermal treatment Ni^2+^ ions could penetrate into the layers of g-C_3_N_4_, thus destroying the van der Waals force between the layers of carbon nitride as the Ni^0^ particles grew. Therefore, the g-C_3_N_4_ underwent a strong structural disruption and rearrangement, then became small particles with layered and porous structures which could greatly enhance the specific surface area of the composite, as well as the amount of exposed active sites for H_2_ evolution. Furthermore, many ball-flower structures were found on composite surface as the Ni precursor content increased to 1.0 and 1.2 (Figure S4). These ball-flower structures are very likely the Ni ball-flower particles, since it was only found when Ni content reached 1.0 and 1.2 (Figure S4e,f).

The elemental mapping images of the chosen area are shown in Figure S5a–e, which demonstrates even distribution of C, N, O and Ni atoms on the 1.0 Ni/CN surface. This indicates that Ni/NiO were evenly distributed on the surface of g-C_3_N_4_. TEM images were further conducted to analyze the surface structure of the Ni/NiO/g-C_3_N_4_ composite (Figure S5f,g). The TEM of 1.0 Ni/CN revealed similar morphology as SEM images show (Figure 4e) that bulk g-C_3_N_4_ locates inside and amorphous Ni/NiO locates outside. Two lattice fringes of 0.203 nm and 0.242 nm were observed in Figure S5g, which are attributed to the Ni (111) crystal plane and NiO (111) crystal plane [23], respectively. Combining the previous XRD patterns (Figure 2a) and XPS spectra (Figure 3) analysis, we can conclude that Ni and NiO species were tightly bounded to each other, and successfully coupled with g-C_3_N_4_.

**Figure 5:**
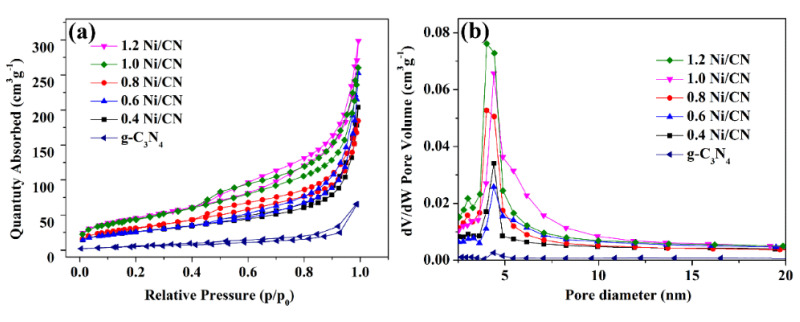
(**a**) N_2_ adsorption/desorption isotherms of the prepared catalysts; (**b**) pore size distribution of all samples.

The nitrogen (N_2_) physisorption isotherms were measured and analyzed to characterize the textural properties of the samples (Figure 5a and Table 1). All the N_2_ adsorption–desorption isotherms exhibit type IV behavior with H3 hysteresis loop [24], indicating the existence of slit-shaped mesopores, which is in accordance with the electron microscopy characterization results (Figure 4 and Figure S4). The Barret–Joyner–Halenda (BJH) pore-size distribution curves are presented in Figure 5b. According to the data in Table 1, the specific surface area of the composite gradually increased from 28.4 to 102 m^2^/g as the Ni content increased from 0 to 0.8, then sharply increased to 148.2 and 152.4 m^2^/g when the Ni content increased to 1.0 and 1.2. The formation of smaller composites with layered and porous structure (Figure 4 and Figure S4) are consider to be the reason for the increase of BET surface areas. The sharp surface area increase of 1.0 Ni/CN and 1.2 Ni/CN are likely due to the Ni ball-flower structure formation, which could disrupt bulk g-C_3_N_4_ more seriously and promote specific surface areas itself.

The pore diameter distribution of catalysts is shown in Figure 5b and the pore volume information is listed in Table 1. The low pore volume of 0.05 m^3^/g of pure g-C_3_N_4_ significantly increased to 0.31–0.46 m^3^/g after Ni introduction. The pore diameter (Figure 5b) also became larger after Ni introduction. Both results are reasonable as the Ni^0^ formation disrupted g-C_3_N_4_ into layered and porous structures.

### 3.4. Optical Properties and Photoelectrochemical Analysis

**Figure 6:**
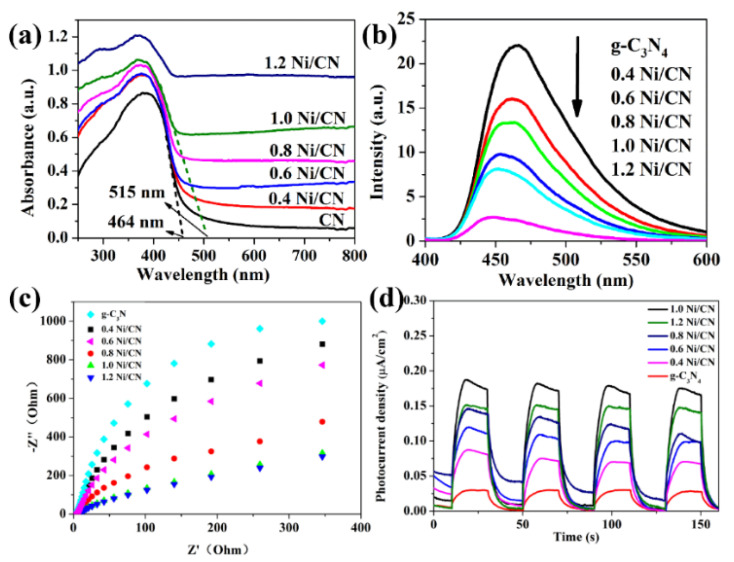
(**a**) UV–vis diffuse reflection spectra and (**b**) photoluminescence spectra (PL) of prepared catalysts; (**c**) electrochemical impedance spectra (EIS) of prepared catalysts and (**d**) transient photocurrent response under visible light irradiation.

The optical properties and the band-edge positions of samples were characterized by UV–vis diffuse reflectance spectra (DRS) as shown in Figure 6a. As Ni content increases, the light absorption intensity and absorption range of samples are greatly enhanced and the light absorption band-edge shifts from 464 nm of g-C_3_N_4_ to 515 nm of 1.0 Ni/CN. The photoluminescence spectrum was used to explore the recombination rate of electrons and holes on catalysts. Photoluminescence test is mainly used to determine the recombination efficiency of photogenerated carriers (Figure 6b). From PL results we can see that, as the Ni loading increases, the recombination of carriers was suppressed. Two possible reasons may account for such a phenomenon: (1) bulk g-C_3_N_4_ was stripped into smaller particles with layered and porous structures (Figure 4 and Figure S4) by the Ni^0^ growth inside g-C_3_N_4_ layers, which could reduce the carrier transfer pathway from the core section of g-C_3_N_4_ to the surface. Therefore, the carrier transfer efficiency from cores to surfaces can be accelerated, and the recombination of electrons and holes can be suppressed. (2) The Ni loading can also accelerate the electron transfer from g-C_3_N_4_ to Ni particles, hence suppressing electron and hole recombination in the composite.

The electrochemical impedance spectra (EIS) can also be used to forecast carrier transfer efficiency by providing charge transfer resistance information of catalysts, which is positive related to the Nyquist curve radius [25]. The Nyquist curve radius decreases with increasing Ni content (Figure 6c), indicating that Ni loading could reduce electrode resistance (i.e., charge transfer resistance) of the composite. A photocurrent measurement is useful to examine carrier separation and transfer efficiency of photocatalysts [26,27]. The photocurrent response intensity increases as the Ni content increases, revealing that Ni loading enhances electron transfer efficiency. We consider that the p–n heterojunction between NiO and g-C_3_N_4_ and the Ni formation as electron receiver are responsible for the increase in carrier separation and migration efficiency. The 1.2 Ni/CN shows lower photocurrent response intensity than 1.0 Ni/CN, likely because overloading of Ni lowers light absorption via a shading effect, hence introducing photo-excited electrons to a lower density in the composite. The PL, EIS, and photocurrent characterizations show an almost similar conclusion that Ni loading could enhance carrier separation and transfer efficiency of the composites.

### 3.5. H_2_ Evolution and Photodegradation Test

**Figure 7:**
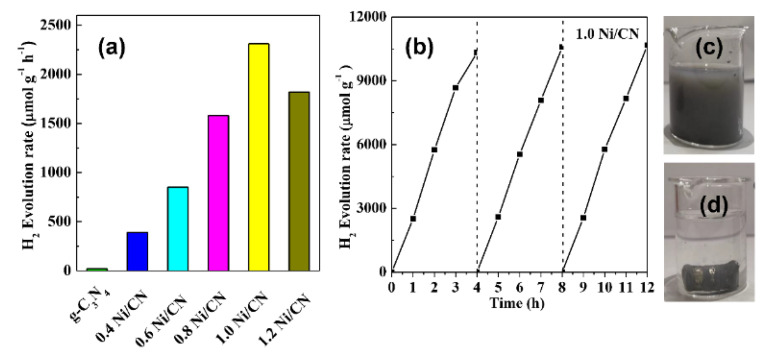
(**a**) H_2_-production of 1.0 Ni/CN, (**b**) recycled photocatalytic H_2_-productionof 1.0 Ni/CN, and (**c**) 1.0 Ni /CN magnetic recycle test image before adding magnet, (**d**) 1.0 Ni /CN magnetic recycle test image after adding magnet.

Figure 7a shows the photocatalytic H_2_-production activities of different samples. The photocatalytic H_2_-production rate is almost negligible for pure g-C_3_N_4_ due to the fast carrier recombination between conduction band (CB) electrons and valence band (VB) holes, as well as the lack of metal sites for H_2_ evolution. As the Ni/CN ratio increases from 0 to 1.0, the H_2_-production rate increased from 20 μmol·h^−1^·g^−1^ of g-C_3_N_4_ to 2310 μmol·h^−1^·g^−1^ of 1.0 Ni/CN. Since NiO/Ni can promote charge separation and transfer efficiency (Figure 6b–d), Ni could act as active sites, and since more surface area can provide more exposed active sites for H_2_ evolution, it is reasonable that the H_2_ evolution capability increases with increasing Ni/NiO content. When the Ni/CN ratio increases from 1.0 to 1.2, the H_2_-production rate decreased to 1820 μmol·h^−1^·g^−1^. We consider that the decreasing Ni^0^ formation (Figure 2a), i.e., the decreasing active sites for H_2_ production, should account for the decrease in H_2_ production. Meanwhile, excessive Ni species could exert a shading effect on g-C_3_N_4_, reducing the light absorption of g-C_3_N_4_.

The cycle experiment and the stability of composites are shown in Figure 7b. After three cycles of the hydrogen production test, the catalyst performance was still stable, proving the excellent stability of the catalysts. Figure 7c and d shows the magnetic recovery test of our catalysts. As shown in Figure 7c, our catalyst can be dispersed in water homogenously; once a magnet is added (Figure 7d), almost all catalysts are immediately attracted to the magnet. This suggests that a catalyst prepared by our method can be easily recovered and reused in practical applications.

We also tested our catalyst in the application of RhB photodegradation (Figure S6). Higher RhB degradation was observed on 1.0 Ni/CN than that on g-C_3_N_4_ and the RhB degradation activity of 1.0 Ni/CN was 1.7 times higher than that of g-C_3_N_4,_ according to the degradation rate constants of each reaction.

### 3.6. Mechanism for Enhanced Photoactivity

**Figure 8:**
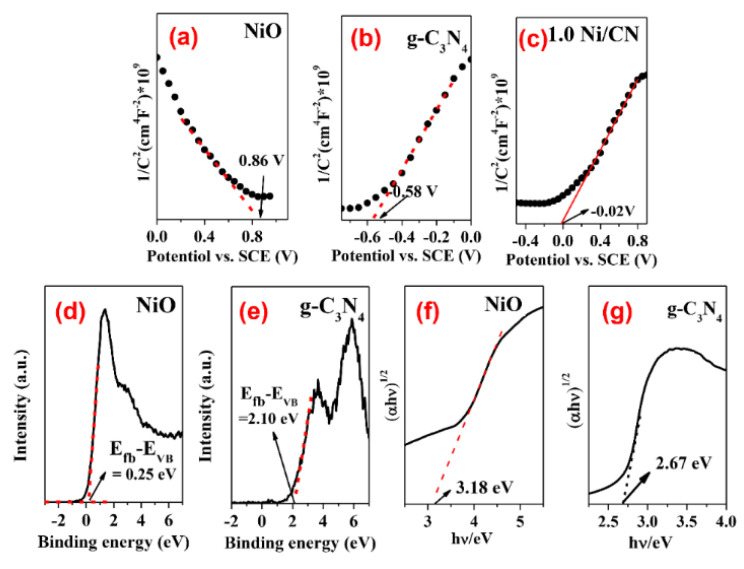
(**a**–**c**) Mott-Schottky plots for pure NiO, g-C_3_N_4,_ and 1.0 Ni/CN; (**d**,**e**) VB-XPS spectra of pure NiO and g-C_3_N_4_; and (**f**,**g**) determination of optical band gap of samples.

In order to explore the mechanism of enhanced carrier separation and transfer efficiency, the energy band structure including band gaps, conduction band (CB), and valence band (VB) positions of pure NiO and bulk g-C_3_N_4_ were determined. The Mott–Schottky curves of NiO and g-C_3_N_4_ are shown in Figure 8. The negative slope of NiO indicates it is a p-type semiconductor (Figure 8a), while the positive slope of g-C_3_N_4_ suggests it is a n-type semiconductor (Figure 8b). As is generally known, the Fermi level (E_fb_) is approximately equal to the plane potential of the semiconductor. The Fermi level (E_fb_) of NiO and g-C_3_N_4_ were calculated to be 0.86 V and −0.58 V versus the Hg/Hg_2_Cl_2_ electrode (Figure 8a,b), which was equal to 1.1 V and −0.34 V versus a normal hydrogen electrode (NHE), respectively. The VB-XPS shows the band gap differences between VB and the Fermi level, i.e., the E_fb_-E_VB_, of NiO and g-C_3_N_4_, which are 0.25 V and 2.10 V, respectively (Figure 8d,e). Based on the E_fb_ and E_fb_-E_VB_ information, we therefore can determine the VB values of NiO and g-C_3_N_4_ are 1.35 eV and 1.76 eV, respectively. Finally, the CB of NiO and g-C_3_N_4_ can be calculated according to their respective band gap values (Figure 8f,g), which are −1.83 eV and −0.91 eV, respectively.

**Scheme 1:**
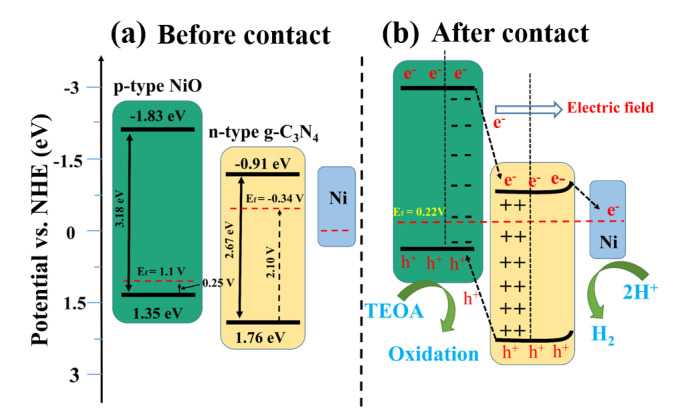
Schematic mechanism of Ni/NiO/g-C_3_N_4_ heterojunction for photocatalytic H_2_ evolution.

Once NiO (p-type) and g-C_3_N_4_ (n-type) are contacted, the electrons of n-type g-C_3_N_4_ will flow to p-type NiO to balance the Fermi energy level until a new equilibrium is reached [28]. In this case, the NiO energy level moves up, and the g-C_3_N_4_ energy level moves down, eventually forming an internal electric field between the semiconductor interfaces. When metallic Ni^0^ is loaded on the surface of g-C_3_N_4_, the energy band of g-C_3_N_4_ is bent [29], resulting in a further Fermi level shift of the ternary complex. Influenced by the above two reasons, the Fermi level of 1.0 Ni/CN finally achieved 0.22 V (Figure 8c, Table 2) and the overall band energy information of 1.0Ni/CN is summarized in Table 1 and Scheme 1.

In this case, due to the existence of the internal electric field, photogenerated electrons will transfer from the NiO to g-C_3_N_4_, subsequently to Ni^0^; while the photogenerated holes on g-C_3_N_4_ will transfer to NiO. The p–n heterojunction between NiO and g-C_3_N_4_ could enhance carrier separation and transfer efficiency. The Ni^0^, on one hand, could take effect as electron receivers are consider to enhance carrier separation and transfer efficiency; on the other hand, could act as active sites for H_2_ evolution.

## 4. Conclusions

In summary, Ni/NiO/CN ternary composites were synthesized by a one-step solvothermal method. Ni and NiO can be simultaneously loaded on g-C_3_N_4_. During the preparation process, the bulk g-C_3_N_4_ disrupted into smaller particles with a layered and porous structure, due to the Ni^0^ growth between g-C_3_N_4_ layers. Consequently, the specific surface area of the composites gradually increased from 28.4 to 148.4 m^2^/g as the Ni precursor content increased from 0 to 1.2, which provided more exposed active sites for H_2_ evolution. The PL, EIS, and photocurrent characterizations reveal similar trends in that increasing Ni loading could increase carrier separation and transfer efficiency, as well as suppress carrier recombination. We consider that the p–n heterojunction between NiO and g-C_3_N_4_ and the Ni formation as electron receiver are responsible for the increase in carrier separation and migration efficiency. The hydrogen evolution rate of 1.0 Ni/CN catalyst shows the highest H_2_ evolution capability of 2310 mol·h^−1^·g^−1^, which is 210 times higher than that of pure g-C_3_N_4_ catalyst.

## Figures and Tables

**Table 1:** Texture properties of prepared samples.

Samples	S_BET_ (m^2^ g^−1^)	Pore Volume (cm^3^ g^−1^)
g-C_3_N_4_	28.4	0.05
0.4 Ni/CN	76.2	0.31
0.6 Ni/CN	82.1	0.36
0.8 Ni/CN	102.6	0.39
1.0 Ni/CN	148.4	0.46
1.2 Ni/CN	152.6	0.41

**Table 2:** Band position and band gap information.

Catalyst	E_fb_ (V vs. SCE)	E_fb_ (V vs. NHE)	Band gap Eg (eV)	E_fd_-E_VB_ (eV)	E_VB_ (V vs. NHE)	E_CB_ (V vs. NHE)
g-C_3_N_4_	−0.58	−0.34	2.67	2.1	1.76	−0.91
NiO	0.86	1.1	3.18	0.25	1.35	−1.83
Ni/NiO/g-C_3_N_4_	−0.02	0.22				

E_fd_(NHE) = E_fb_(SCE) + 0.241; E_VB_ = E_fb_ + (E_fb_ − E_VB_); E_CB_ = E_VB_ – Eg.

## Data Availability

Data sharing not applicable.

## Supplementary Materials

The following are available online at https://www.mdpi.com/article/10.3390/ma14112894/s1, Figure S1: ATR-FTIR spectra of prepared samples, Figure S2: Ni^2+^ adsorption and reduction mechanism, Figure S3: Energy Dispersive Spectroscopy (EDS) pattern of (a) pure g-C_3_N_4_; (b) 0.4 Ni/CN; (c) 0.6 Ni/CN; (d) 0.8 Ni/CN; (e) 1.0 Ni/CN and (f) 1.2 Ni/CN, Figure S4: Zoom-in view of SEM image of (a) pure g-C_3_N_4_; (b) 0.4 Ni/CN; (c) 0.6 Ni/CN; (d) 0.8 Ni/CN; (e) 1.0 Ni/CN and (f) 1.2 Ni/CN, Figure S5: (a) elemental mapping images of (b) C, (c) N, (d) O, (e) Ni in 1.0 Ni/CN; (f), (g) TEM and HRTEM of 1.0 Ni/CN, Figure S6: (a) Photocatalytic degradation of RhB over pure bulk CN, and 1.0 Ni/CN photocatalysts. (b)The degradation rate constant of RhB over bulk CN, and 1.0 Ni/CN photocatalysts samples, Table S1: Summary of papers on Ni species and carbon nitride composites in recent years, Table S2: The elemental composition and content of all samples.
