# Supplementary material for: Electronegativity Assisted Synthesis of Magnetically Recyclable Ni/NiO/g-C3N4 for Significant Boosting H2 Evolution
Source: Materials (Basel). 2021 May 28;14(11):2894. doi: 10.3390/ma14112894 (PMC8199054; doi:10.3390/ma14112894)
Supplement: Supplementary file 1 [file materials-14-02894-s001.zip › materials-1190050-supplementary.pdf]

materials

Supplementary Materials

Electronegativity Assisted Synthesis of Magnetically Recyclable Ni/NiO/g-C_3N_4 for Significant Boosting H_2 Evolution

Tingfeng Zhang [1], Ping Liu [1], Lili Wang [1], Shuai Wang [2], Jinsheng Shi [1] and Xuefang Lan [1,*]

1 Department of Chemistry and Pharmacy, Qingdao Agricultural University, Qingdao 266000, China; ztf824066980@163.com (T.Z.); pingliu@qau.edu.cn (P.L.); liliwang@qau.edu.cn (L.W.); cdws@163.com (S.W.); jsshiqn@aliyun.com (J.S.)
2 Chengyang branch of Qingdao Ecological Environment Bureau, Qingdao 266000, China
* Correspondence: xuefanglan@hotmail.com

Table S1. Summary of papers on Ni species and carbon nitride composites in recent years.

Catalysts	synthetic method	Light source	Activity ($\mu molg^{-1}h^{-1}$)	Recycling performance	Reference
Ni/NiO/g-C_3N_4	solvothermal method (ethonal)	300 W Xe-lamp/300 W Xe-lamp (λ>420 nm)	2310	yes	This work
NiO/g-C_3N_4	high temperature calcination	300 W Xe-lamp (λ>420 nm)	68.8	no	[1]
Ni_xP_y/g-C_3N_4	hydrothermal method	500 W Xe-lamp ($\lambda \geq$420 nm)	162	no	[2]
Ni/NiO/g-C_3N_4	high temperature hydrogen reduction	300 W Xe-lamp (λ>420 nm)	10	no	[3]
Ni/g-C_3N_4	solvothermal method (DMF)	500 W Xe-lamp	313.2	no	[4]
Ni/g-C_3N_4	solvothermal method (DMF)	500 W Xe-lamp	168.2	no	[5]
$Ni(OH)_2$/g-C_3N_4	hydrothermal method	150 W Xe lamp (200 -2500 nm)	95.4	no	[6]
WO_3/g-C_3N_4/ $Ni(OH)_x$	photodeposition method	300 W Xe-lamp(λ>400 nm)	576	no	[7]
NiS_2/g-C_3N_4	electroless deposition	Four LEDs (3 W, 420 nm)	116.343	no	[8]
NiS/g-C_3N_4	high temperature calcination	350 W Xe-lamp(λ>420 nm)	36.87	no	[9]
2D-$Ni(OH)_2$/3D-g-C_3N_4	electroless deposition	300 W	87.2	no	[10]

Ni-P/g-C_3N_4	electroless deposition	Xe-lamp 350 W Xe-lamp	937	no	[11]
Ni_2P/g-C_3N_4	High temperature phosphating process	300 W Xe-lamp(λ>420 nm)	82.5	no	[12]
PtNi/g-C_3N_4	chemical reduction method	300 W Xe-lamp(λ>420 nm)	104.7	no	[13]
NiS/g-C_3N_4	hydrothermal method	300 W Xe-lamp(λ>400 nm)	482	no	[14]
$Ni_{12}P_5$/g-C_3N_4	mechanical grinding method	350 W Xe-lamp(λ>420 nm)	126.61	no	[15]
NiS_2/g-C_3N_4	hydrothermal method	300 W Xe-lamp(λ>420 nm)	715.83	no	[16]
NiO/g-C_3N_4	wetness impregnation method	300 W Xe-lamp(λ>420 nm)	90.05	no	[17]
$Ni_{12}P_5$/g-C_3N_4	two-step solution-phase method	300 W Xe-lamp(λ>420 nm)	535.7	no	[18]
NiS_2 Quantum Dots/g-C_3N_4	hydrothermal method	300 W Xe-lamp(λ>420 nm)	968.2	no	[19]
NiS/g-C_3N_4	ion-exchange method	300 W Xe-lamp (λ>420 nm)	447.7	no	[20]
Ni/NiS/g-C_3N_4	high temperature hydrogen reduction	300 W Xe-lamp (λ≥420 nm)	515	no	[21]
Ni_2P/g-C_3N_4	low-temperature phosphidation method	300 W Xe-lamp (λ>420 nm)	567	no	[22]
NiCoP/g-C_3N_4	High temperature phosphating process	300 W Xe-lamp	1643	no	[23]

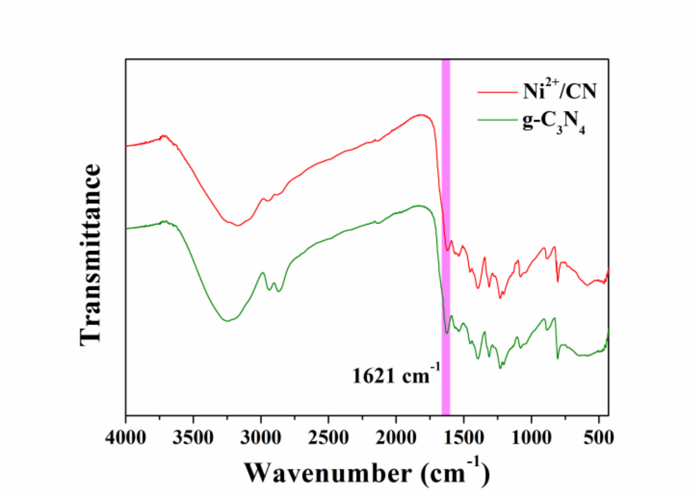

Figure S1. ATR-FTIR spectra of prepared samples.

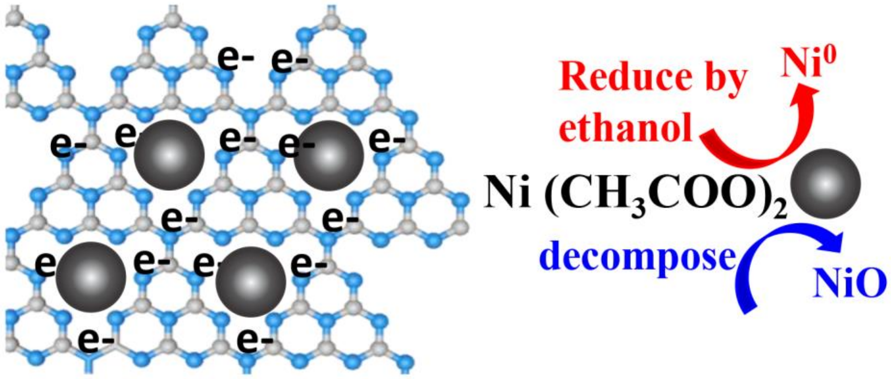

Figure S2. Ni^{2+} adsorption and reduction mechanism.

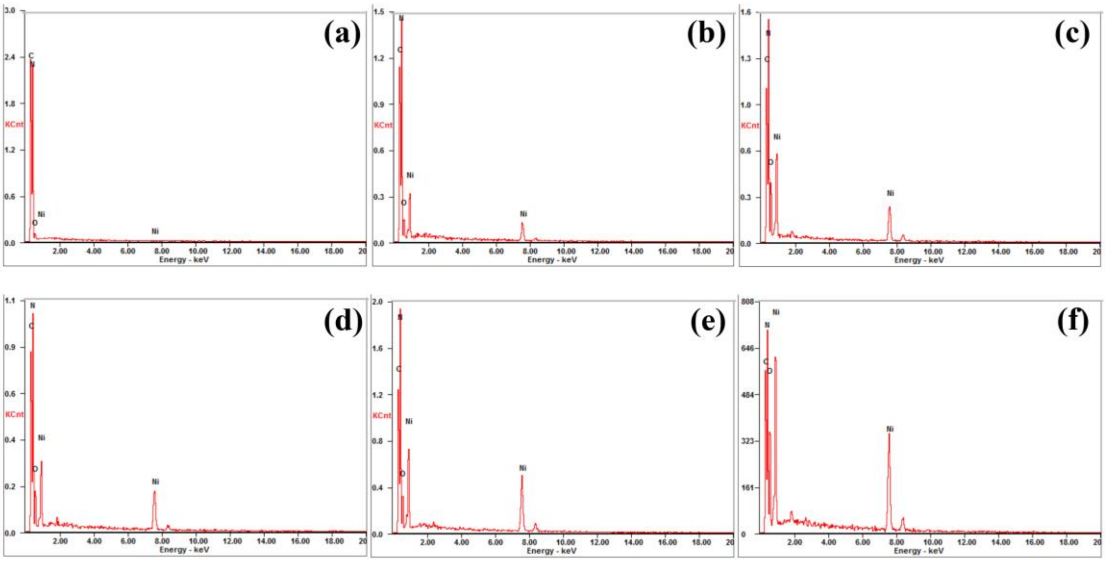

Figure S3. Energy Dispersive Spectroscopy (EDS) pattern of (a) pure g-C_3N_4; (b) 0.4 Ni/CN; (c) 0.6 Ni/CN; (d) 0.8 Ni/CN; (e) 1.0 Ni/CN and (f) 1.2 Ni/CN.

Table S2. The elemental composition and content of all samples.

Catalyst	Ni (wt%)	C (wt%)	N (wt%)	O (wt%)
g-C_3N_4	0	34	44.16	04.32
0.4 Ni/CN	09.57	31.16	50.00	06.27
0.6 Ni/CN	14.55	29.08	38.67	07.34
0.8 Ni/CN	18.65	29.22	40.57	9.68
1.0Ni/CN	23.72	28.25	37.64	11.40
1.2 Ni/CN	29.93	29.45	39.76	14.55

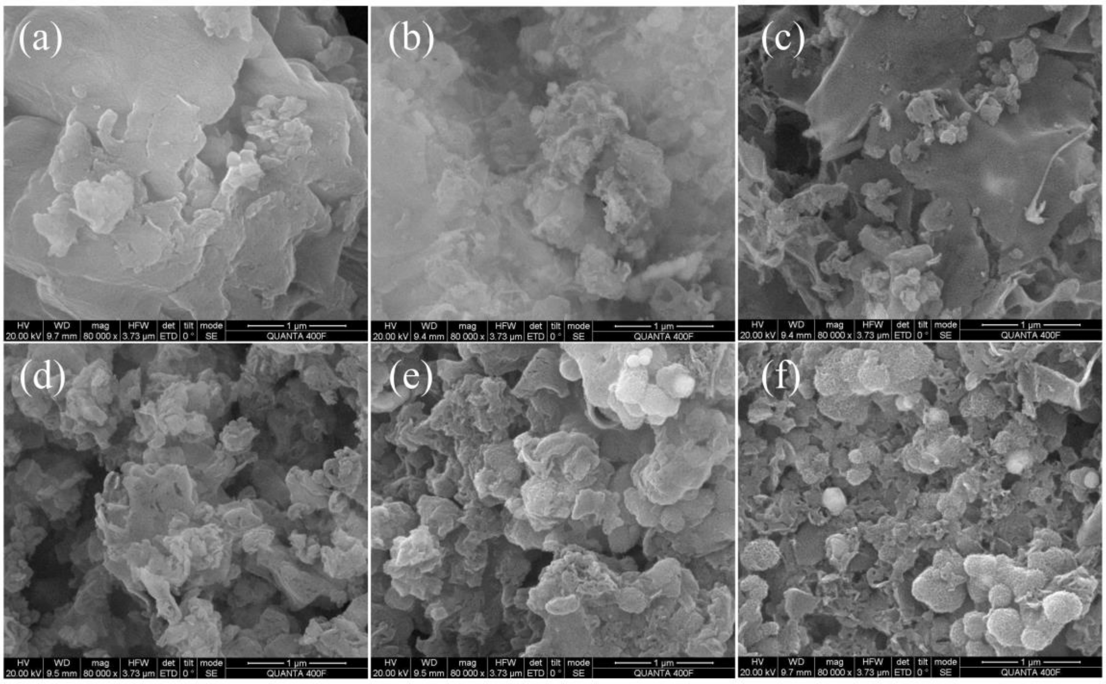

Figure S4. Zoom-in view of SEM image of (a) pure g- C_3N_4; (b) 0.4 Ni/CN; (c) 0.6 Ni/CN; (d) 0.8 Ni/CN; (e) 1.0 Ni/CN and (f) 1.2 Ni/CN.

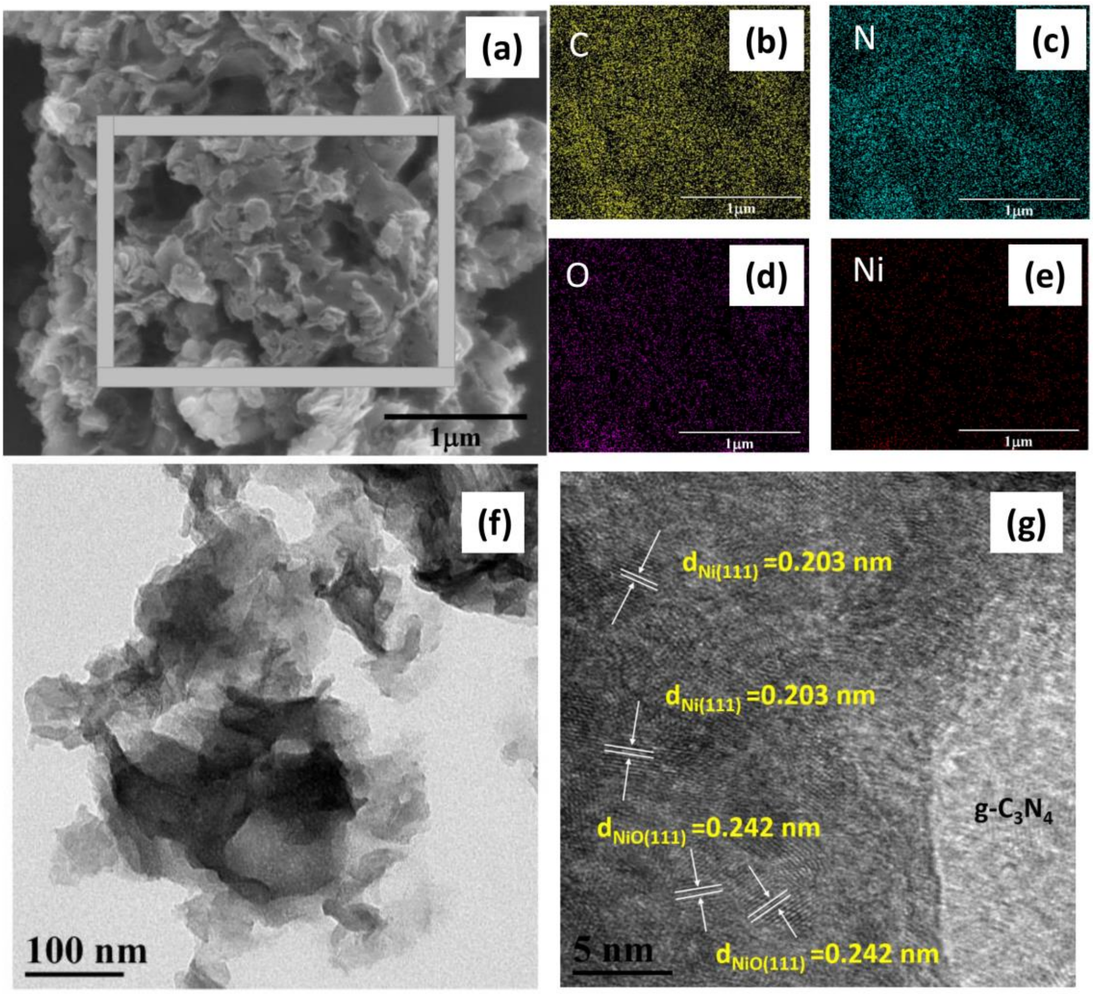

Figure S5. (a) elemental mapping images of (b) C, (c) N, (d) O, (e) Ni in 1.0 Ni/CN; (f), (g) TEM and HRTEM of 1.0 Ni/CN.

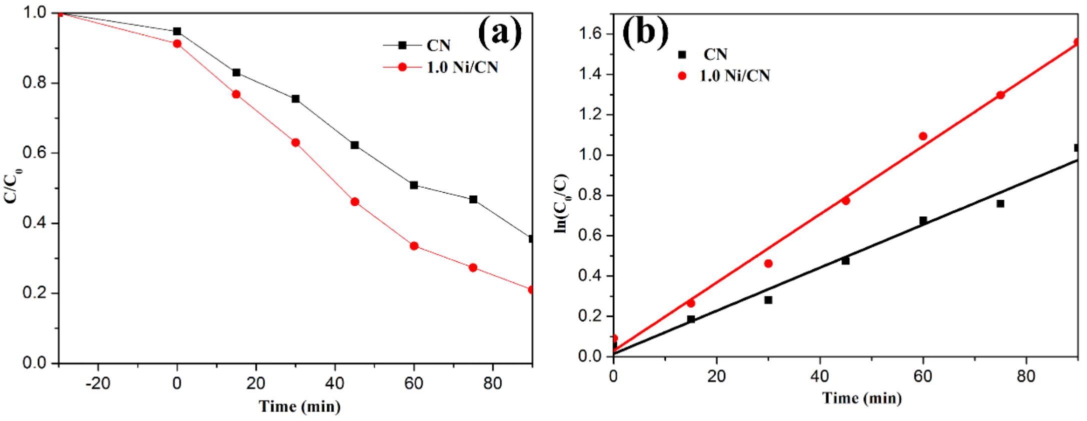

Figure S6. (a) Photocatalytic degradation of RhB over pure bulk CN, and 1.0 Ni/CN photocatalysts. (b)The degradation rate constant of RhB over bulk CN, and 1.0 Ni/CN photocatalysts samples.

References

1. Liu, J.; Jia, Q.; Long, J.; Wang, X.; Gao, Z.; Gu, Q., Amorphous NiO as co-catalyst for enhanced visible-light-driven hydrogen generation over g-C3N4 photocatalyst. *Applied Catalysis B: Environmental* **2018,** 222, 35-43.
2. Sun, Z.; Zhu, M.; Fujitsuka, M.; Wang, A.; Shi, C.; Majima, T., Phase Effect of NixPy Hybridized with g-C3N4 for Photocatalytic Hydrogen Generation. *ACS Appl Mater Interfaces* **2017**, 9, (36), 30583-30590.
3. Zhang, G.; Li, G.; Wang, X., Surface Modification of Carbon Nitride Polymers by Core-Shell Nickel/Nickel Oxide Cocatalysts for Hydrogen Evolution Photocatalysis. *ChemCatChem* **2015,** 7, (18), 2864-2870.
4. Bi, L.; Meng, D.; Bu, Q.; Lin, Y.; Wang, D.; Xie, T., Electron acceptor of Ni decorated porous carbon nitride applied in photocatalytic hydrogen production. *Phys Chem Chem Phys* **2016,** 18, (46), 31534-31541.
5. Bi, L.; Xu, D.; Zhang, L.; Lin, Y.; Wang, D.; Xie, T., Metal Ni-loaded g-C3N4 for enhanced photocatalytic H2 evolution activity: the change in surface band bending. *Phys Chem Chem Phys* **2015,** 17, (44), 29899-905.
6. Yan, J.; Wu, H.; Chen, H.; Pang, L.; Zhang, Y.; Jiang, R.; Li, L.; Liu, S., One-pot hydrothermal fabrication of layered β-Ni(OH) 2 /g-C 3 N 4 nanohybrids for enhanced photocatalytic water splitting. *Applied Catalysis B: Environmental* **2016,** 194, 74-83.
7. He, K.; Xie, J.; Luo, X.; Wen, J.; Ma, S.; Li, X.; Fang, Y.; Zhang, X., Enhanced visible light photocatalytic H 2 production over Z-scheme g-C 3 N 4 nansheets/WO 3 nanorods nanocomposites loaded with Ni(OH) x cocatalysts. *Chinese Journal of Catalysis* **2017,** 38, (2), 240-252.
8. Chen, F.; Yang, H.; Wang, X.; Yu, H., Facile synthesis and enhanced photocatalytic H 2 -evolution performance of NiS 2 -modified g-C 3 N 4 photocatalysts. *Chinese Journal of Catalysis* **2017,** 38, (2), 296-304.
9. He, K.; Xie, J.; Li, M.; Li, X., In situ one-pot fabrication of g-C 3 N 4 nanosheets/NiS cocatalyst heterojunction with intimate interfaces for efficient visible light photocatalytic H 2 generation. *Applied Surface Science* **2018,** 430, 208-217.
10. Cao, R.; Yang, H.; Zhang, S.; Xu, X., Engineering of Z-scheme 2D/3D architectures with Ni(OH)2 on 3D porous g-C3N4 for efficiently photocatalytic H2 evolution. *Applied Catalysis B: Environmental* **2019,** 258.
11. Qi, K.; Xie, Y.; Wang, R.; Liu, S.-y.; Zhao, Z., Electroless plating Ni-P cocatalyst decorated g-C3N4 with enhanced photocatalytic water splitting for H2 generation. *Applied Surface Science* **2019,** 466, 847-853.
12. Ye, P.; Liu, X.; Iocozzia, J.; Yuan, Y.; Gu, L.; Xu, G.; Lin, Z., A highly stable non-noble metal Ni2P co-catalyst for increased H2 generation by g-C3N4 under visible light irradiation. *Journal of Materials Chemistry A* **2017,** 5, (18), 8493-8498.
13. Peng, W.; Zhang, S.-S.; Shao, Y.-B.; Huang, J.-H., Bimetallic PtNi/g-C3N4 nanotubes with enhanced photocatalytic activity for H2 evolution under visible light irradiation. *International Journal of Hydrogen Energy* **2018,** 43, (49), 22215-22225.
14. Hong, J.; Wang, Y.; Wang, Y.; Zhang, W.; Xu, R., Noble-metal-free NiS/C3 N4 for efficient photocatalytic hydrogen evolution from water. *ChemSusChem* **2013,** 6, (12), 2263-8.
15. Wen, J.; Xie, J.; Shen, R.; Li, X.; Luo, X.; Zhang, H.; Zhang, A.; Bi, G., Markedly enhanced visible-light photocatalytic H2 generation over g-C3N4 nanosheets decorated by robust nickel phosphide (Ni12P5) cocatalysts. *Dalton Transactions* **2017,** 46, (6), 1794-1802.
16. Lv, C.; Lan, X.; Wang, L.; Yu, Q.; Zhang, M.; Sun, H.; Shi, J., Alkaline-earth-metal-doped TiO2 for enhanced photodegradation and H2 evolution: insights into the mechanisms. *Catalysis Science & Technology* **2019,** 9, (21), 6124-6135.
17. Shi, H.; Bi, J.; Bai, C.; Wu, J.; Xu, Y.; Han, Y.; Zhang, X., Nickel Ammine Complex-derived NiO Modified g-C3N4

Composites with Enhanced Visible-light Photocatalytic H2 Evolution Performance. *ChemistrySelect* **2019,** 4, (27), 8095-8103.

18. Zeng, D.; Ong, W.-J.; Zheng, H.; Wu, M.; Chen, Y.; Peng, D.-L.; Han, M.-Y., Ni12P5 nanoparticles embedded into porous g-C3N4 nanosheets as a noble-metal-free hetero-structure photocatalyst for efficient H2 production under visible light. *Journal of Materials Chemistry A* **2017,** 5, (31), 16171-16178.
19. Xue, F.; Liu, M.; Cheng, C.; Deng, J.; Shi, J., Localized NiS2 Quantum Dots on g-C3N4 Nanosheets for Efficient Photocatalytic Hydrogen Production from Water. *ChemCatChem* **2018,** 10, (23), 5441-5448.
20. Chen, Z.; Sun, P.; Fan, B.; Zhang, Z.; Fang, X., In Situ Template-Free Ion-Exchange Process to Prepare Visible-Light-Active g-C3N4/NiS Hybrid Photocatalysts with Enhanced Hydrogen Evolution Activity. *The Journal of Physical Chemistry C* **2014,** 118, (15), 7801-7807.
21. Wen, J.; Xie, J.; Zhang, H.; Zhang, A.; Liu, Y.; Chen, X.; Li, X., Constructing Multifunctional Metallic Ni Interface Layers in the g-C3N4 Nanosheets/Amorphous NiS Heterojunctions for Efficient Photocatalytic H2 Generation. *ACS Appl Mater Interfaces* **2017,** 9, (16), 14031-14042.
22. Zhao, H.; Sun, S.; Jiang, P.; Xu, Z. J., Graphitic C 3 N 4 modified by Ni 2 P cocatalyst: An efficient, robust and low cost photocatalyst for visible-light-driven H 2 evolution from water. *Chemical Engineering Journal* **2017,** 315, 296-303.
23. Bi, L.; Gao, X.; Zhang, L.; Wang, D.; Zou, X.; Xie, T., Enhanced Photocatalytic Hydrogen Evolution of NiCoP/g-C3 N4 with Improved Separation Efficiency and Charge Transfer Efficiency. *ChemSusChem* **2018,** 11, (1), 276-284.
